# Inactivation of Glutamine Synthetase-Coding Gene *glnA* Increases Susceptibility to Quinolones Through Increasing Outer Membrane Protein F in *Salmonella enterica* Serovar Typhi

**DOI:** 10.3389/fmicb.2020.00428

**Published:** 2020-03-20

**Authors:** Ana R. Millanao, Aracely Y. Mora, Claudia P. Saavedra, Nicolás A. Villagra, Guido C. Mora, Alejandro A. Hidalgo

**Affiliations:** ^1^Escuela de Química y Farmacia, Facultad de Medicina, Universidad Andres Bello, Santiago, Chile; ^2^Instituto de Farmacia, Facultad de Ciencias, Universidad Austral de Chile, Valdivia, Chile; ^3^Facultad de Ciencias Químicas y Farmacéuticas, Universidad de Chile, Santiago, Chile; ^4^Laboratorio de Microbiología Molecular, Departamento de Ciencias Biológicas, Facultad de Ciencias de la Vida, Universidad Andres Bello, Santiago, Chile; ^5^Millennium Institute on Immunology and Immunotherapy, Departamento de Ciencias Biológicas, Facultad de Ciencias de la Vida, Universidad Andres Bello, Santiago, Chile; ^6^Escuela de Tecnología Médica, Universidad Andres Bello, Santiago, Chile; ^7^Departamento de Laboratorios Clínicos, Escuela de Medicina, Pontificia Universidad Católica de Chile, Santiago, Chile; ^8^Instituto de Investigación Interdisciplinar en Ciencias Biomédicas SEK, Facultad de Ciencias de la Salud, Santiago, Universidad SEK, Santiago, Chile

**Keywords:** *glnA*, ciprofloxacin, OmpF, *S*. Typhi, transposon

## Abstract

Ciprofloxacin is the choice treatment for infections caused by *Salmonella* Typhi, however, reduced susceptibility to ciprofloxacin has been reported for this pathogen. Considering the decreased approbation of new antimicrobials and the crisis of resistance, one strategy to combat this problem is to find new targets that enhances the antimicrobial activity for approved antimicrobials. In search of mutants with increased susceptibility to ciprofloxacin; 3,216 EZ-Tn*5* transposon mutants of *S*. Typhi were screened. *S.* Typhi *zxx*::EZ-Tn*5* mutants susceptible to ciprofloxacin were confirmed by agar diffusion and MIC assays. The genes carrying EZ-Tn*5* transposon insertions were sequenced. Null mutants of interrupted genes, as well as inducible genetic constructs, were produced using site-directed mutagenesis, to corroborate phenotypes. SDS-PAGE and Real-time PCR were used to evaluate the expression of proteins and genes, respectively. Five mutants with increased ciprofloxacin susceptibility were found in the screening. The first confirmed mutant was the glutamine synthetase-coding gene *glnA*. Analysis of outer membrane proteins revealed increased OmpF, a channel for the influx of ciprofloxacin and nalidixic acid, in the *glnA* mutant. Expression of *ompF* increased four times in the *glnA* null mutant compared to WT strain. To understand the relationship between the expression of *glnA* and *ompF*, a strain with the *glnA* gene under control of the tetracycline-inducible P^tet^ promoter was created, to modulate *glnA* expression. Induction of *glnA* decreased expression of *ompF*, at the same time that reduced susceptibility to ciprofloxacin. Expression of sRNA MicF, a negative regulator of OmpF was reduced to one-fourth in the *glnA* mutant, compared to WT strain. In addition, expression of *glnL* and *glnG* genes (encoding the two-component system NtrC/B that may positively regulate OmpF) were increased in the *glnA* mutant. Further studies indicate that deletion of *glnG* decreases susceptibility to CIP, while deletion of *micF* gene increases susceptibility CIP. Our findings indicate that *glnA* inactivation promotes *ompF* expression, that translates into increased OmpF protein, facilitating the entry of ciprofloxacin, thus increasing susceptibility to ciprofloxacin through 2 possible mechanisms.

## Introduction

*Salmonella* Typhi is the etiological agent of typhoid fever, endemic in many developing countries worldwide. This disease is exclusive of humans with a mortality rate of 10%. The emergence of the extensively drug-resistant *S.* Typhi H58 strains in Pakistan, which are resistant to the first-line drugs (ampicillin, chloramphenicol, and cotrimoxazole), fluoroquinolones, and third-generation cephalosporin, is a real threat with potential to become typhoid fever untreatable (Cabello, 2018; Johnson et al., 2018; Klemm et al., 2018). Thus, typhoid fever requires alternative pharmacological treatment, including new antimicrobials and development of new combined therapies to revitalize existing antibiotics to prolong its useful life and slow down the emergence of resistance (Cottarel and Wierzbowski, 2007; Fajardo et al., 2008; Liu et al., 2010; Rodas et al., 2010; Sabbagh et al., 2012; González-Bello, 2017).

To find mutants with increased susceptibility to Ciprofloxacin (CIP), a screening was performed over 3,216 insertional mutants of *S*. Typhi STH2370 with the EZ-Tn*5*^TM^ transposon exposed to sub-MIC concentrations of CIP. We identified both: a small number of known and new genetic determinants of resistance to quinolones. One such mutant in the *glnA* gene that encodes for glutamine synthetase (GS) was further characterized. GS produces glutamine from glutamate and ammonia and has a crucial role in nitrogen metabolism. The internal concentration of glutamine is the main intracellular signal for regulating nitrogen availability in enteric bacteria (Zimmer et al., 2000; Switzer et al., 2018). Nitrogen is essential for the biosynthesis of macromolecules in bacteria; thus, the adaptive response to metabolic stress induced by starvation of nitrogen (as it is the case of glutamine auxotrophic bacteria) could affect bacterial physiology, including the susceptibility to antimicrobials which today is known to be modulated by metabolism (Maria-Neto et al., 2012; Peng et al., 2015; Vestergaard et al., 2017; Cui et al., 2019).

One example that relates nitrogen metabolism and susceptibility to antimicrobials is the observation of *Escherichia coli* strains resistant to magainin I (a cationic peptide) which overexpress GS (Maria-Neto et al., 2012). In *Streptococcus pneumoniae* it was demonstrated that *glnA* was repressed in the presence of penicillin, as well as, the inhibition of GS enhanced susceptibility to penicillin. Hence, glutamine conferred a protective role against penicillin when added to the culture medium (El Khoury et al., 2017). In the same line, methicillin-resistant *Staphylococcus aureus* and methicillin-susceptible *S. aureus* with lower expression of GS decreased their level of methicillin resistance (Gustafson et al., 1994). It is proposed that in *S. aureus* GS participates in the production of constituents of the cell envelope, therefore maintaining the cell wall thickness and the level of crosslinking on peptidoglycan (Gustafson et al., 1994; Lima et al., 2013). In *Mycobacterium tuberculosis*, GS plays a crucial role in the cell wall biosynthesis; therefore, inhibition of GS in *M. tuberculosis* with the inhibitor L-methionine-*SR*-sulfoximine increases susceptibility to isoniazid. Again, this study concludes that inhibition of GS activity affects the synthesis of peptidoglycan layers of the cell wall, changing susceptibility (Harth and Horwitz, 2003).

In this work, we describe that *glnA* mutants of *S.* Typhi increase their susceptibility to quinolones. Interestingly OmpF, a porin forming a homotrimer channel for the influx of CIP and nalidixic acid, was augmented in the *S*. Typhi *glnA* mutant. Further studies using a tetracycline-inducible system, revealed an inverse correlation between *glnA* and *ompF* expression. Our findings suggested that *glnA* inactivation increases the susceptibility of *S.* Typhi to CIP through increasing OmpF.

## Materials and Methods

### Bacterial Strains and Growth Conditions

*Salmonella* Typhi SHT2370 an antibiotic-sensitive virulent clinical strain was obtained from the Infectious Diseases Hospital Lucio Cordova, Santiago, Chile (Valenzuela et al., 2014). Mutants used in this study were derived from *S*. Typhi STH2370 and are shown in Table 1. Bacteria were grown in LB medium (1% peptone, 0.5% yeast extract, 0.5% NaCl) at 37°C with aeration. When required, the medium was supplemented with Kanamycin (KAN) 50 mg/L, Ampicillin (AMP) 100 mg/L, Chloramphenicol (CAM), CIP (at different concentrations) or Chlortetracycline (CTET at different concentrations). Media were solidified with agar (15 g/L) as required. Mutants in genes *glnL* and *glnG* were produced by P22 transducing null mutations with the KAN^R^ cassette from *S*. Typhimurium.

**TABLE 1:** Strains and plasmids used in this work.

Strain	Genotype/relevant features	Source
STH2370	*Salmonella* Typhi strain	Lab. stock
STH2370 *glnA::*EZ-Tn*5*	*glnA::*EZ-Tn*5*	This study
STH2370 *glnA::kan*	Δ*glnA*::*kan*	This study
STH2370 *glnA*::FRT	Δ*glnA*::FRT	This study
SHT2370 *glnA*^TD^	ΔP^glnA^::[*tetR-*P^tet^-Δ*tetA*::FRT]	This study
STH2370 *glnG::kan*	Δ*glnG*::*kan*	This study
STH2370 *glnL::kan*	Δ*glnL*::*kan*	This study
STH2370 *micF*::*cam*	Δ*micF*::*cam*	This study
STH2370 *ompF*::*kan*	Δ*ompF*::*kan*	This study
STH2370 *ompF*^TD^	ΔP^ompF^::[*tetR-*P^tet^-Δ*tetA*::FRT]	This study
STH2370 STY4173*^TD^*	ΔP^4173^::[*tetR-*P^tet^-Δ*tetA*::FRT]	This study
*E*. *coli* DH5αλpir	encodes pir protein of phage lambda, used for propagation of plasmids with R6K replication origin	Lab. stock
Plasmid		
pKD4	AMP^R^, KAN^R^, FRT-KAN^R^-FRT	Datsenko and Wanner, 2000
pKD46	AMP^R^, λ Red Recombination System	Datsenko and Wanner, 2000
pCP20	AMP^R^, Flp expression	Datsenko and Wanner, 2000

### Transposon Insertional Mutagenesis and Screening

Mutagenesis with the commercial transposon EZ-Tn*5*^TM^ (Epicentre, CA, United States) was performed as indicated by the provider (protocols available at https://www.lucigen.com/docs/manuals/MA155E-EZ-Tn5-r6kg -Ori-KAN-2-Transposome-Kit.pdf at February 17, 2020) with minor modifications. In brief, *S.* Typhi STH2370 was grown to OD_600_ 0.4, 5 mL were washed six times with sterile water, resuspended in 100 μL of sterile water and electroporated with 1 μL of EZ-Tn*5* transposome at a concentration of 0.1 pmol/μL. Electroporated bacteria were recovered in 1 mL of LB broth, and incubated with aeration for 40 min before plating on LB-agar containing KAN 50 mg/L and overnight incubation. Mutants *S*. Typhi mutants STH2370 *zxx*::EZ-Tn*5* were organized on a 6 × 8 pattern in 67 lots creating a collection of 3,216 mutants. The primary screening was performed by transferring colonies to LB plates with CIP 0.05 mg/L using a 48-pins microplate replicator. Mutants with increased susceptibility were selected by visual examination and confirmed by disk diffusion assay. The susceptibility of confirmed mutants was quantified by MICs assays before proceeding with cloning and sequencing of interrupted genes.

### Cloning and Sequencing

The mutants with at least two-fold-change in the MIC were subjected to cloning. Briefly, genomic DNA of mutants was extracted, digested with *Eco*RV and ligated with T4 DNA ligase. Ligated DNA was electroporated in *E*. *coli* DH5αλpir, recovered in 1 mL of LB broth, and incubated with aeration for 40 min at 37°C before plating on LB-agar containing KAN 50 mg/L. KAN^R^ colonies were grown, and plasmidial DNA was column-purified to perform Sanger sequencing. Sequences were analyzed using the Basic Local Alignment Search Tool available at http://www.ncbi.nlm.nih.gov.

### Disk Diffusion Test

The antibiotic susceptibility tests were performed by Kirby-Bauer disk diffusion method according to CLSI guidelines (CLSI, 2012a). Suspensions of 2 mL of microorganism were grown overnight in liquid culture using LB medium with aeration. The bacterial suspensions were diluted 1:1000 in 0.9% NaCl and 100 μL of this suspension were seeded on Mueller Hinton (MH) agar plates pH 7.3. CIP susceptibilities tests were performed with 5 μg CIP disks onto seeded plates incubated at 37°C for 16–18 h. Halos were recorded as the average of 2-crossed diameters in biological triplicate assays.

### Determination of MIC

MIC was determined using broth microdilution susceptibility testing according to CLSI guidelines (CLSI, 2012b). Briefly, 0.9% NaCl was used to prepare inoculum until turbidity McFarland 0.5, with an equivalent density to 1–2 × 10^8^ CFU/mL. The inoculum was then diluted to reach a concentration of 5 × 10^4^ CFU/well. Antibiotics were serially diluted in base-2 and each well-inoculated with 50 μL of diluted bacteria. The plates were incubated at 37°C for 16 h and read at 600 nm. The MIC was determined as the concentration that allowed less than 50% growth density compared to the non-antibiotic control.

### Construction of *S.* Typhi Site-Directed Mutants

Site-directed mutants of *S*. Typhi STH2370 were constructed by allelic exchange facilitated by the Red/Swap Technique (Datsenko and Wanner, 2000). Briefly, PCR product, with a resistance cassette flanked by regions with homology to the target DNA sequences, was electroporated into strains carrying pKD46, recovered in 1 mL of LB broth and incubated for 1 h at 37°C with aeration before plating onto plates with the corresponding antibiotic to select mutants. When needed the resistance cassette was eliminated to produce FRT scar mutants by introducing pCP20 which encodes the Flippase recombinase that promotes scission from FRT sequences flanking the resistance cassette. The primers used for these constructions are in Supplementary Table 1.

### Construction of Tetracycline-Dependent Mutants

Construction of the tetracycline-dependent (TD) mutants *S.* Typhi *glnA*^TD^, *S.* Typhi *ompF*^TD^ and *S.* Typhi STY4173^TD^, was carried out using a modification of the Red-Swap method for promoter mutation of target genes as described by Hidalgo et al. (2016). The primers used for these constructions are in Supplementary Table 1.

### Preparation of Outer Membrane Proteins (OMPs) and SDS-PAGE

The OMPs fraction of protein was obtained by a method previously described (Lobos and Mora, 1991). Two mL of bacteria were centrifuged at 10,000 *g* for 10 min. The pellet obtained was resuspended in 10 mM Tris Hydrochloride pH 8, sonicated for 100 s and centrifuged at 7,000 *g* for 5 min. The supernatant obtained was centrifuged at 13,000 *g* for 45 min, and the pellet was resuspended in 10 mM Tris Hydrochloride pH 8 with 2% Triton X-100. The suspension was incubated at 37°C for 30 min with occasional stirring and centrifuged at 13,000 *g* for 45 min. The pellet containing OMPs was resuspended in 100 mM Tris Hydrochloride pH 8, 2% SDS and quantified using BCA protein assay kit. Next, 50 μg of protein were heated at 98°C for 5 min and resolved in an SDS-PAGE at 100 V. The gels were rinsed with water, pre-treated for 60 min with 40% ethanol and 10% acetic acid, washed twice for 10 min and stained in 0.1% acid Coomassie blue R250, 2% orthophosphoric acid, 10% ammonium sulfate, and 20% methanol overnight. Finally, gels were washed with 1% acetic until all Coomassie particles were removed. High resolution pictures of gels were open in the ImageJ software. Using the area selection tool, a horizontal line was traced to cut pick that were then analyzed by their density, which was compared to density of WT strain for the bands corresponding to OmpF (Gallo-Oller et al., 2018).

### RNA Isolation, Reverse Transcription, and Real-Time PCR (RT-qPCR)

For extraction of RNA, overnight cultures were diluted 100 times and cultured in fresh LB or LB + CTET as appropriate, until reaching OD_600_ 0.5. RNA was extracted using the Trizol method according to the provider instructions. Briefly, 20 mL of bacterial cell was harvested, washed twice with ice-cold water and treated with lysozyme at RT before adding Trizol^TM^ (Invitrogen). The samples were mixed before adding ice-cold chloroform, centrifugation and recovering the supernatant. RNA was precipitated with isopropanol, washed with 70% v/v ethanol and resuspended in nuclease-free water before treatment with DNase I. Reverse transcriptions of samples were performed with 1 μg of DNase-treated RNA using SuperScript^TM^ II Reverse Transcriptase (Invitrogen) with Random Hexamers (Invitrogen). Primers for the genes *dnaN* and *lon* were used to normalize the expression of target genes. Although *dnaN* and *lon* are both good expression controls, *dnaN* was preferred for assays that used tetracyclines to minimize distortion of results as *dnaN* remained unchanged compared to *lon*, in presence of CTet. Quantification was carried out in an Eco^TM^ Real-Time PCR System using Brilliant II SYBR^TM^ Master Mixes. The fold change was calculated as described by Pfaffl (Pfaffl, 2001). The primers used for these assays are listed in Supplementary Table 1.

### Statistical Analysis

Student’s *t*-test was realized if samples obtained of different experiments have passed Shapiro–Wilk test for normality. The data are presented as mean ± SD of at least biological triplicates. Statistical analyses of results from RT-qPCR were calculated using the paired Student’s *t*-test (GraphPad Prism version 8.0, San Diego, CA, United States).

## Results

### Screening of *S.* Typhi *zxx*::EZ-Tn5 to Identify Mutants With Increased Susceptibility to Ciprofloxacin

We screened 3,216 ***S***. Typhi STH2370 ***zxx***::EZ-Tn***5*** mutants on MH-agar plates with CIP 0.05 mg/L. A total of 43 susceptible mutants with poor or not growth were selected by visual analysis of the colonies. Confirmation of CIP susceptible mutants was performed by using disk diffusion assay. From this test, 10 mutants were confirmed, as their inhibition halos were 6 mm greater compared with WT control and were subjected to MIC assays. Eight mutants were twice as sensitive, one mutant was four-times more sensitive, and one mutant was eight-times more sensitive to CIP, compared with the WT counterpart. These mutants were set for cloning and further sequencing of the interrupted genes. All selected mutants that were sent to sequencing had one transposon insertion only. An extensive search was performed for information linking the mutated genes to antibiotics resistance. Five mutants, *recC*::EZ-Tn*5, cysE*::EZ-Tn*5*, *aefA*::EZ-Tn*5*, *dacC*::EZ-Tn*5* and *glnA*::EZ-Tn*5*, resulted of especial interest after considering two criteria: (i) the level of susceptibility of the mutant and (ii) the relevance/novelty of the interrupted gene. Supplementary Figure 1 shows the screening workflow to identify genes leading to susceptibility. Subsequently, a secondary screening was performed, over the five mutants of interest, to determine whether observed susceptibility was general or specific for one antimicrobial or a family of them. Diameters of inhibition haloes for the five insertional mutants selected with different antimicrobials were recorded (Supplementary Table 2). After discarding the best candidate (*S*. Typhi *aefA*::EZ-Tn*5*) whose increased susceptibility was product of polar effects produced by the transposon over the *acrAB* locus upstream of *aefA*gene, we began the characterization of mutant*S*. Typhi STH2370 *glnA::*EZ-Tn*5* that showed increased susceptibility to NAL and CIP in the screening (Supplementary Table 2). While the other three mutants were not further characterized.

### *S.* Typhi *glnA* Mutants Are More Susceptible to Ciprofloxacin

**FIGURE 1:**
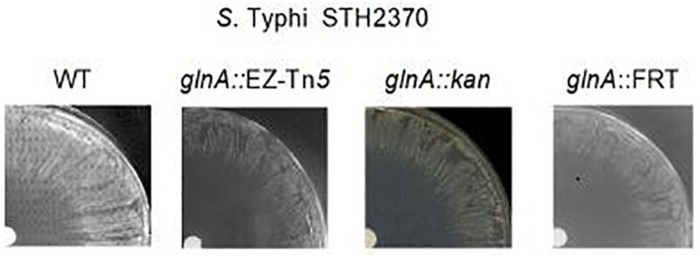
*Salmonella* Typhi mutants in *glnA* gene show susceptibility to ciprofloxacin. A disk diffusion assay was performed to measure inhibition zones of *S.* Typhi STH2370 (WT), *S.* Typhi STH2370 *glnA*::EZ-Tn*5*, *S.* Typhi STH2370 *glnA*::*kan* and *S.* Typhi STH2370 *glnA*::FRT. The strains were seeded onto MH agar plates pH 7.3. Followed, 5 μL of ciprofloxacin 2 mg/mL were spotted onto a filter paper disk and placed on the plates and incubated at 37°C for 16–18 h.

To rule out polar effects that might modulate antimicrobial susceptibility, in the *S.* Typhi *glnA*::EZ-Tn*5*, we constructed mutant *S.* Typhi STH2370 *glnA*::*kan* and *S.* Typhi STH2370 *glnA*::FRT (Supplementary Figure 2). All mutants in *glnA* gene (*glnA*::EZ-Tn*5*, *glnA*::*kan*, and *glnA*::FRT) presented the same range of increased susceptibility to CIP with a diameter of inhibition haloes ∼11 mm larger and a MIC twice as sensitive compared to the WT strain (Figure 1 and Table 2). The observation that *S*. Typhi *glnA*::FRT (as well as all null mutants in *glnA* gene) shows the same level of susceptibility, rule out any possible polar effect governing the observed phenotype, as FRT sequence is by far less invasive that resistance cassettes.

**TABLE 2:** Susceptibility to ciprofloxacin of WT *S.* Typhi and null mutants in *glnA*.

Strain	Inhibition haloes (mm ± SD)	MIC mg/L ± SD	MIC compared to control
*S.* Typhi STH2370	37.3 ± 1.25	0.078 ± 0	Control
*glnA::*EZ-Tn*5*	47.8 ± 0.76	0.039 ± 0	1/2 CIM
*glnA::kan*	48.0 ± 0.50	0.039 ± 0	1/2 CIM
*glnA::*FRT	48.2 ± 0.29	0.039 ± 0	1/2 CIM

### Inactivation of *glnA* Gene Increases the Expression of OmpF

**FIGURE 2:**
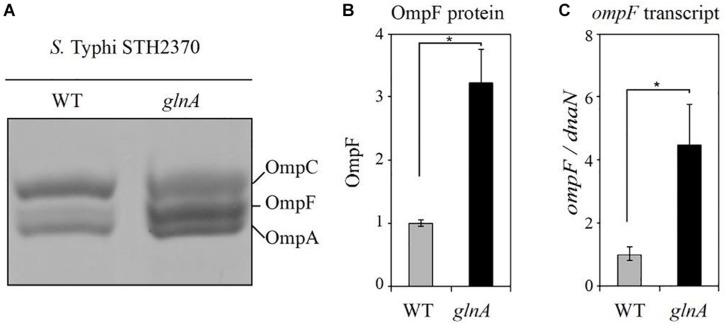
The *glnA* mutant has increased OmpF. **(A)** OMPs profile from *S*. Typhi STH2370 (WT) and *S*. Typhi STH2370 *glnA::*FRT (*glnA*). **(B)** Densitometric analysis of the OmpF porin in the *glnA* mutant compared with WT strain. Analysis was performed by using the ImageJ software, as described in Section “Materials and Methods.” **(C)** Expression level of *ompF* transcripts in the *glnA* mutant compared with the WT strain. Expression of *ompF* transcripts was normalized to expression of *dnaN* and compared to the expression of *ompF* in the WT strain. The figure shows the mean of three assays. Asterisks indicate significance, calculated by Student’s *t*-test, of results linked by brackets ^∗^*p* ≤ 0.05.

Because inactivating *glnA* gene increases susceptibility to CIP, we searched for information regarding determinants of susceptibility to this antibiotic. As outer membrane proteins may be implicated in antibacterial entry through the cell envelope (Nikaido, 2003), we decided to study the profiles of OMPs in the *glnA*::FRT. SDS-PAGE profile of OMPs, showed a threefold increase of OmpF in *S.* Typhi STH2370 *glnA*::FRT, compared with WT (Figures 2A,B). Same result was observed for all *glnA* null mutants in *S.* Typhi, the results for *S.* Typhi *glnA*::FRT and *S.* Typhi *glnA*::*kan* are shown in Supplementary Figure 3, compared to *S.* Typhi WT and a *S.* Typhi *ompF* mutant. Interestingly, deletion of *ompF* produces changes in the OMPs pattern, at the level of making OMPs unrecognizable by simple Coomassie staining. Previous observation reported changes on the amount of OMPs in an *ompF* null mutant of a clinical isolate of *S*. Typhi. However, in this mutant main porins, such as *ompC* and *ompA* did not change their migration pattern (Villarreal et al., 2014). It could be the case that the extend of changes produced after *ompF* is mutated differs between isolates of *S*. Typhi. Despite that, abundance of OmpF would be the responsible of increased susceptibility to CIP, as *ompF* is also augmented in the *ompF* inducible mutant, but not other porins, as shown in further experiments. Next, RT-qPCR was performed to measure *ompF* expression in *glnA*::FRT mutant. As shown in Figure 2C, the expression of *ompF* gene was at least four-times higher in the *glnA*::FRT mutant compared with WT strain.

### Induction of *glnA* Inversely Correlates With Susceptibility to Ciprofloxacin

To confirm how changes in *glnA* expression cause changes in *ompF* expression and susceptibility to CIP, we modulated the expression of *glnA* by using a tetracycline-inducible system as previously described (Hidalgo et al., 2016). For that purpose, we successfully replaced the *glnA* gene promoter with the *tetRA* cassette (Supplementary Figure 4). This mutant allows modulation of *glnA* expression by using tetracyclines, to study *ompF* expression and susceptibility to CIP as a function of *glnA* expression. Because both OmpF and TetA (the efflux pump for tetracyclines) participate in transport through membranes, we excised *tetA* gene, from the *tetRA* cassette, to reduce interferences, caused by the presence of TetA in the inner membrane. This last strain was named as the tetracycline-dependent *glnA* mutant (or simply *glnA*^TD^), as shown in Supplementary Figure 4. Using the same strategy, we constructed the tetracycline-dependent *ompF* mutant (*ompF*^TD^), and the same inducible construct was produced for the unrelated gene STY4173 (4173^TD^) that encodes for a putative protein, as a control. Details regarding these mutants are shown in Supplementary Figure 5. Using the *S.* Typhi *glnA*^TD^ mutant, we observed that inhibition haloes in the presence of CIP decreased, as the concentration of CTET increases in the plates, to values comparable to the inhibition diameters observed for the WT strain (from 46.0 mm without induction to 38.1 mm with 0.05 μg/mL of CTET). In addition, in uninduced conditions, the *glnA*^TD^ mutant shows an inhibition diameter closer to *glnA*::FRT null mutant, 46.0 mm and 48.0 mm, respectively (Table 3).

**TABLE 3:** Induction of *glnA* in *glnA*^TD^ mutant decreases the susceptibility to ciprofloxacin (CIP).

	Diameter of inhibition haloes in presence of CIP (mm) ± SD				
CTET	*glnA*^TD^	*ompF*^TD^	STY4173^TD^	WT	*glnA*::FRT
vehicle (water)	46.0 ± 0.10	31.9 ± 0.25	40.1 ± 0.12	37.0 ± 0.17	48.0 ± 0.17
0.025 μg/mL	41.9 ± 0.17	37.3 ± 0.00	40.0 ± 0.10	38.9 ± 0.42	49.1 ± 0.25
0.050 μg/mL	38.1 ± 0.42	41.0 ± 0.17	40.0 ± 0.10	39.1 ± 0.5	48.8 ± 0.44

In contrast, using the *ompF*^TD^ mutant, the inhibition haloes in the presence of CIP increased as the concentration of CTET increases in the plates, to values close to the inhibition diameters observed for the WT strain (from 31.9 mm without induction to 41.0 mm in the plates with 0.05 μg/mL of CTET). Induction of STY4173^TD^ expression did not cause significant changes in the inhibition halo to CIP (Table 3). The treatment with CTET did not produce significant changes in susceptibility of either WT or *glnA*::FRT strain to CIP.

### Expression of *glnA* Inversely Correlates With *ompF* Expression

**FIGURE 3:**
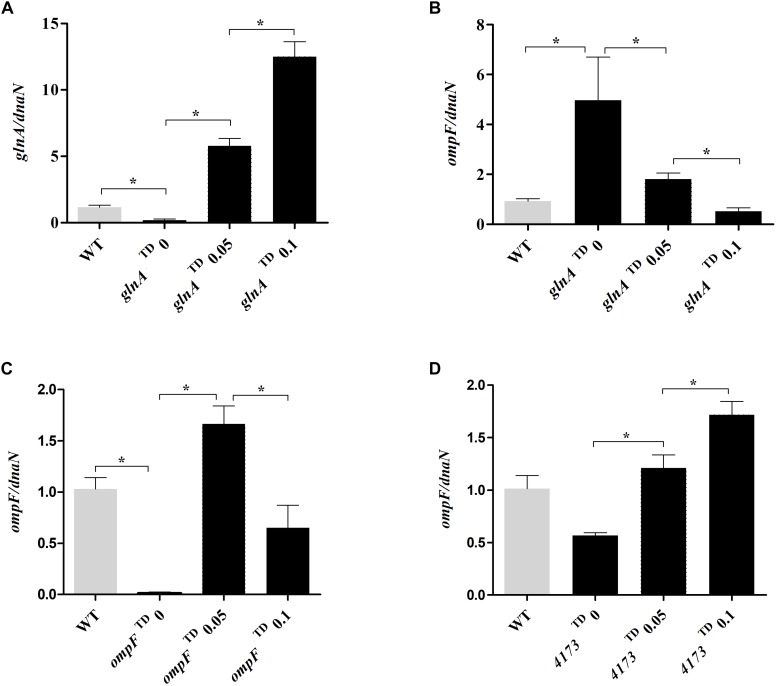
Expression of *ompF* and its dependency in *glnA* expression. Expression level of *glnA* transcripts in the mutant *glnA*^TD^ **(A)** and expression of *ompF* transcripts in the mutant *glnA*^TD^ **(B)**, *ompF*^TD^ **(C)** and *4173*^TD^ **(D)**. The mRNA was extracted from tetracycline-dependent mutants under induced (0.05–0.1 μg/mL CTET) and uninduced conditions. Expression of *glnA* or *ompF* was normalized to expression of *dnaN* and compared to expression of *glnA* or *ompF* in the WT strain without induction. Results are average of at least three biological replicates. Asterisks indicate significance, calculated by Student’s *t*-test, of results liked by brackets. *p ≤ 0.05.

In the view of the decreased susceptibility to CIP when *glnA* is induced with CTET, we decided to assess the expression of *glnA* and *ompF* transcripts in the tetracycline-dependent mutants by RT-qPCR. Expression of *glnA* was increased as CTET concentrations increased (Figure 3A). Next, we investigated the expression of *ompF* in the *glnA*^TD^ with and without CTET induction. We found that in the *glnA*^TD^ mutant without induction, *ompF* is overexpressed close to five-times compared with WT strain (Figure 3B), the expression of *ompF* in the *glnA*^TD^ mutant without induction, is similar to the expression of *ompF* observed in *S*. Typhi *glnA*::FRT null mutant (Figure 2C). When *glnA* expression was induced with CTET we observed a significant decrease in *ompF* expression. Using the *ompF*^TD^ mutant, we found that without induction, expression of *ompF* is dramatically reduced compared to WT strain. Induction of *ompF*^TD^ mutant with 0.05 μg/mL of CTET significantly induced expression of *ompF* over 150-times compared to uninduced condition and over 1.5-times compared to the WT strain. Higher concentration (0.1 μg/mL) induced *ompF* only 50 times compared to uninduced condition and produces only half the expression of *ompF* observed in WT strain (Figure 3C). As a control, we measured the induction of *ompF* after inducing the unrelated gene STY4173 (Figure 3D). The presence of CTET induced expression of *ompF* two to threefold compared to uninduced condition and was slightly higher compared to WT strain in the presence of 0.1 μg/mL CTET. The results indicate that expression of *glnA* inversely correlates with expression of *ompF*, while increased expression of *ompF* may be responsible of increased susceptibility to CIP.

### Inactivation of *glnA* Reduces Expression of MicF

**FIGURE 4:**
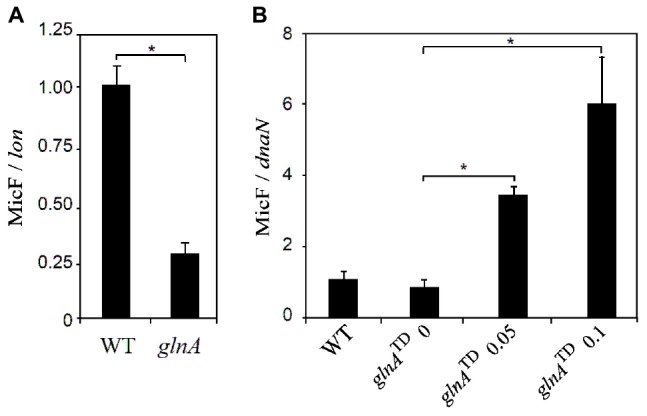
Expression of MicF transcripts depends on expression of *glnA*::FRT. Expression of MicF was studied in a *glnA* null mutant and in the *glnA*^TD^ mutant with different concentrations of chlortetracycline (0, 0.05, or 0.1 μg/mL). Expression of MicF was normalized to the expression of *lon* **(A)** or *dnaN* **(B)** and compared to the expression of MicF in the WT strain. Results are average of at least three biological replicates. Asterisks indicate significance, calculated by Student’s *t*-test, of results linked by brackets. *p ≤ 0.05.

MicF is a small RNA that promotes degradation of *ompF* transcript and blocks translation by interacting with the Shine-Dalgarno sequence (Morillon, 2018). Because of this direct effect of MicF over OmpF, expression of MicF sRNA was studied in the *glnA* mutant and in the *glnA*^TD^ mutant with different levels of *glnA* expression. As observed in the Figure 4A, the *S.* Typhi *glnA*::FRT null mutant expresses around one-fourth of MicF, compared to the *S.* Typhi WT. To corroborate this result, MicF expression was studied in the *S.* Typhi *glnA*^TD^ mutant. Increased expression of *glnA* with 0.05 and 0.1 μg/mL CTET (as shown in Figure 3A) parallel with increased expression of MicF (Figure 4B). It is also observed that in uninduced conditions, MicF levels are slightly reduced compared with WT strain, probably MicF expression is sensitive to low levels of GlnA that transcribes even in the absence of induction with CTET. Conversely, deletion of *micF* gene slightly increased susceptibility to CIP in *S.* Typhi (Table 4). MicF is a well-described sRNA that negatively regulates transcript levels of *ompF*. Whether the induction of MicF by GlnA is direct or through other factors is unknown.

**TABLE 4:** Susceptibility to ciprofloxacin of *S.* Typhi null mutants in genes *glnL*, *glnG*, *micF, and ompF.*

*S*. Typhi STH2370	Inhibition haloes (mm ± SD)
*Wild type*	36.4 ± 0.4
Δ*glnL::kan*	36.2 ± 0.0
Δ*glnG::kan*	32.6 ± 0.8
Δ*micF::cam*	39.1 ± 0.3
Δ*ompF::kan*	27.7 ± 0.3

### The Deletion of *glnA* Activates Transcription of NtrBC Two-Component System

**FIGURE 5:**
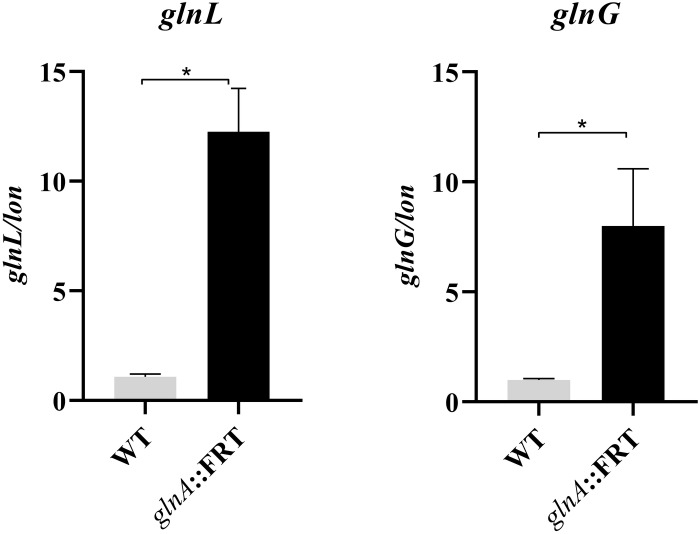
Expression level of *glnL* and *glnG* transcripts in the mutant *glnA*::FRT compared to WT strain. Expression of transcripts was normalized to the expression of *lon* and compared to the expression of *glnL* or *glnG* in the of WT strain. Results are average of at least three biological replicates. Asterisks indicate significance, calculated by Student’s *t*-test, of results liked by brackets. *p ≤ 0.05.

In *E. coli* previous findings indicate that *ompF* is under the control of NtrBC two-component system encoded by *glnL and glnG*, as observed in microarray assays (Zimmer et al., 2000). In enterobacteria, these genes are found downstream of *glnA* and are involved in the stress response of enteric bacteria to nitrogen-limited growth (Bhagirath et al., 2019) (Supplementary Figure 2). The deletion of *glnA* increases the expression of *glnL* and *glnG*, 12- and 8-times respectively, compared to *S.* Typhi WT (Figure 5), indicating that *glnA* negatively regulates expression of *glnL* and *glnG* through maintaining steady concentrations of glutamine. Mutating *glnA* reveals a positive regulation of NtrBC on the expression of *ompF*. This regulation pathway may underly the mechanism of increased susceptibility to CIP in the *glnA* mutants. To study the phenotype associated to *glnL* and *glnG*, null mutations of these genes were transduced into *S.* Typhi to test for resistance to CIP. As shown in Table 4, deletion of *glnL* gene has no effect on resistance to CIP. When *glnG*, which encodes for a transcriptional regulator, is deleted in *S*. Typhi, the mutant shows moderately, yet significantly, resistant to CIP. It is plausible that while over expression of GlnG correlates with susceptibility, deletion of glnG produces resistance. However, the correlation of activation of NtrBC two-component system [encoded by glnL (NtrB) and glnG (NtrC) genes] need to be further validated.

## Discussion

The threat of extensively drug-resistant *S.* Typhi isolates requires the development of new treatment strategies. One approach is the search for antibiotics adjuvants-compounds (Murima et al., 2014; Baquero and Martínez, 2017). In a non-exhaustive screening of *S.* Typhi STH2370, we found the mutant *S*. Typhi *glnA*::EZ-Tn*5*, with half the MIC to CIP. This change reveals a low-level resistance, mediated by an intrinsic mechanism of protection in a microorganism that is in principle susceptible to CIP. It is thought that the genes determining such intrinsic low-levels of resistance (2- to 4-times the MIC) might be of clinical importance and they could be target for adjuvant therapies (Baquero and Martínez, 2017). GS plays a crucial role in nitrogen metabolism whose main function is to provide glutamine to the cell. In Enterobacteria, the reaction catalyzed by GS is the only biosynthetic pathway for glutamine synthesis; thus, *glnA* deletion results in glutamine auxotrophy (Ikeda et al., 1996; Shimizu, 2013). The *glnA* gene has not been previously related with changes in susceptibility to quinolones, in Gram-negative bacteria. Expression of the major porin OmpF, a well-characterized channel for CIP and NAL entry, was increased across all *glnA* null mutants tested and the inducible *glnA* mutant without induction.

Interestingly, the mechanism behind increased susceptibility differs from affecting integrity of the cell wall, as antimicrobials targeting the cell wall have little or no effect on the *glnA* null mutant (Supplementary Table 2). Our results contrast with previous reports regarding GS and resistance to antimicrobials (Harth and Horwitz, 2003; Lima et al., 2013; El Khoury et al., 2017). It was found an inverse correlation between expression of *glnA* and *ompF* and a direct correlation between expression of *glnA* and *micF*. Decreased expression of *micF* in the *glnA* null mutant might explain at least in part the increased expression of *ompF* and increased susceptibility to CIP in the *glnA* mutants.

**FIGURE 6:**
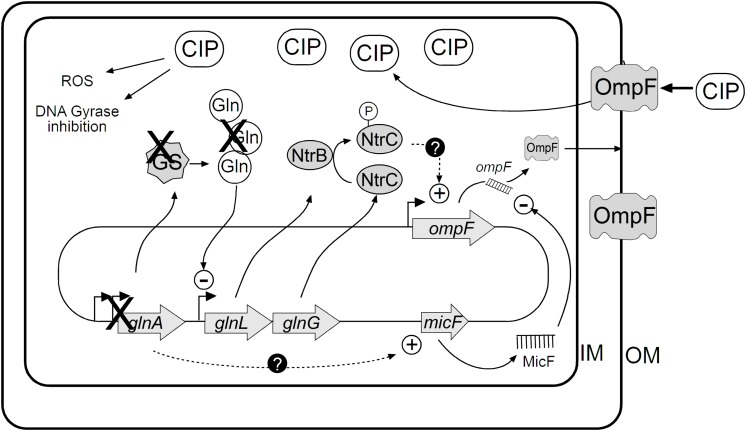
Integrative model of the regulation of OmpF and its implication in susceptibility to CIP. Expression of OmpF is negatively controlled by GlnA. Proposed model illustrating how *glnA* gene is necessary to maintain low expression of OmpF. Based in our data, deleting *glnA* gene promotes *ompF* expression that in turns facilitates CIP and nalidixic acid entry into the bacterial cell through OmpF.

The growth under nitrogen-limiting conditions activates the Nitrogen regulation stress response, under the control of NtrBC two-component system encoded by *glnL* (NtrB) and *glnG* (NtrC) genes. This two-component system is composed of NtrB, the cognate histidine kinase that phosphorylates NtrC to its active form to regulate expression of genes including *glnA*, as previously described (van Heeswijk et al., 2013). In this study, inactivation of *glnA* resulted in increased expression of *glnL* and *glnG* genes. Importantly, it was found that expression of *ompF* is controlled by NtrC in *E. coli* (Zimmer et al., 2000). Therefore, this increase in the expression of *glnL* and *glnG* may explain the increased OmpF and increased susceptibility when *glnA* expression is suppressed or reduced as we illustrate in Figure 6. Indeed, mutation of *glnG* (encoding for NtrC transcriptional regulator) produces moderate resistance to CIP. This observation is consistent with increased *glnLG* expression in the *glnA* mutants, which may trigger *ompF* expression, finally allowing entry of CIP through OmpF porin.

Other mechanisms, modulating antimicrobial resistance were studied including regulators MarA, MarR, and RamA; efflux-system components AcrB and TolC and porin OmpC. We found no differences in the expression of the genes encoding for such antimicrobial’s resistance factors (Supplementary Figure 6). Thus, enhanced susceptibility to quinolones observed in the *glnA* mutants could be linked to the increase of OmpF and associated with nutritional stress due to the decrease of glutamine and lower expression of MicF.

## Conclusion

The experimental results show that the inactivation of *glnA* gene drives to increased susceptibility to CIP in *S.* Typhi, through increasing outer membrane protein OmpF. The finding that genes implicated in nitrogen metabolism, such as *glnA*, modulate susceptibility to antimicrobials can be useful in search of potential targets to combat antimicrobial resistance.

## Data Availability Statement

The datasets generated for this study are available on request to the corresponding author.

## Author Contributions

ARM and AH participated in the conception of ideas, performed the experiments, analyzed and interpreted the data, and participated in the drafting of manuscript. AYM performed experiments, collected and analyzed the data, and revised the draft. CS, NV, and GM collected and analyzed the data and participated in drafting of manuscript.

## Conflict of Interest

The authors declare that the research was conducted in the absence of any commercial or financial relationships that could be construed as a potential conflict of interest.
