# Supplementary material for: Inactivation of Glutamine Synthetase-Coding Gene glnA Increases Susceptibility to Quinolones Through Increasing Outer Membrane Protein F in Salmonella enterica Serovar Typhi
Source: Front Microbiol. 2020 Mar 20;11:428. doi: 10.3389/fmicb.2020.00428 (PMC7103639; doi:10.3389/fmicb.2020.00428)
Supplement: Supplementary file 1 [file Data_Sheet_1.PDF]

Supplementary Material

1 Supplementary Figures and Tables

1.1 Supplementary Figures

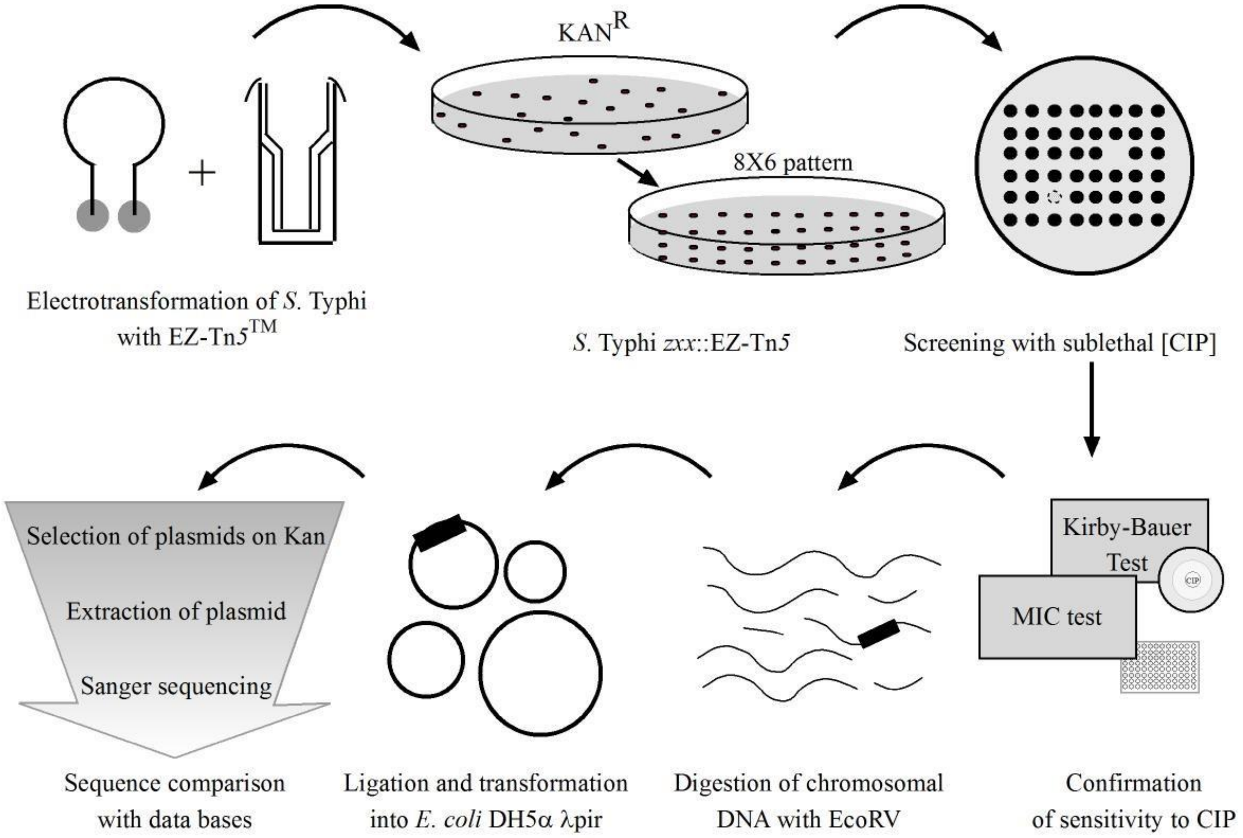

Supplementary Figure 1. Workflow of the screening with sublethal concentrations of ciprofloxacin using the mutants *S.* Typhi STH2370 *zxx*::EZ-Tn*5*.

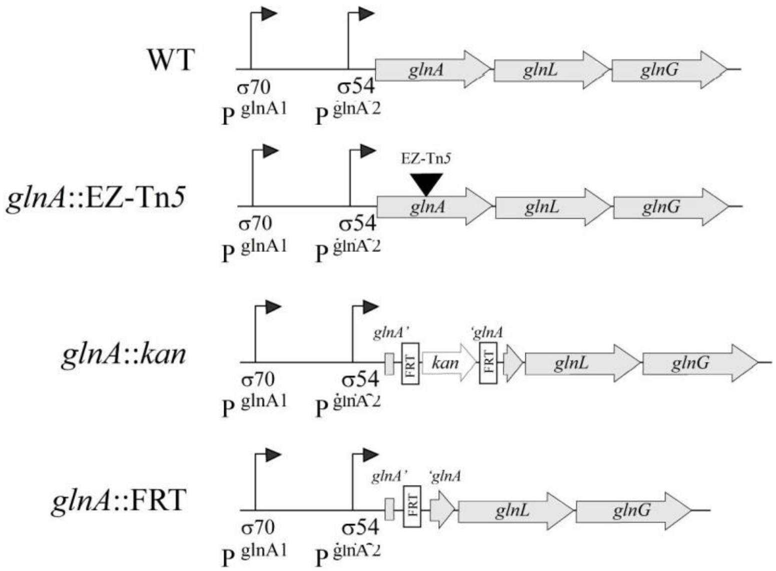

Supplementary Figure 2. The *glnA* operon of WT *S*. Typhi and the *glnA* null mutants used in this study**.** The diagram shows the WT: *S.* Typhi *glnA* genetic context; the *glnA*::EZ-Tn*5*: insertional mutant with EZ-Tn*5*™ transposon; the *glnA*::*kan*: *glnA* deletion with kanamycin cassette; and the *glnA*::FRT: *glnA* deletion without resistance. Length of genes and promoters are not at scale.

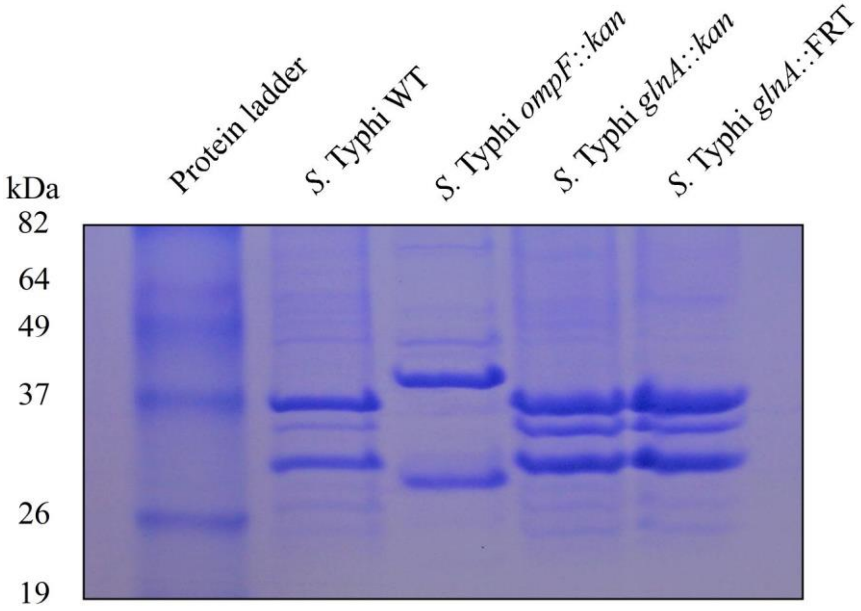

Supplementary Figure 3. 12.5 % SDS-PAGE of OMP from *S*. Typhi WT, *S*. Typhi *ompF*, S. Typhi *glnA*::*kan* and *S.* Typhi *glnA*::FRT. OMP extracts were obtained from bacterial cultures grown overnight in LB medium at 37°C. 50 μg of protein were boiled 5 minutes at 98°C in loading buffer and resolved in 12.5 % SDS-PAGE and stained with Coomassie blue. OmpF is augmented in both *glnA* mutants analysed, compared with *S*. Typhi WT. Deletion of OmpF produce dramatic changes in the pattern of porins.

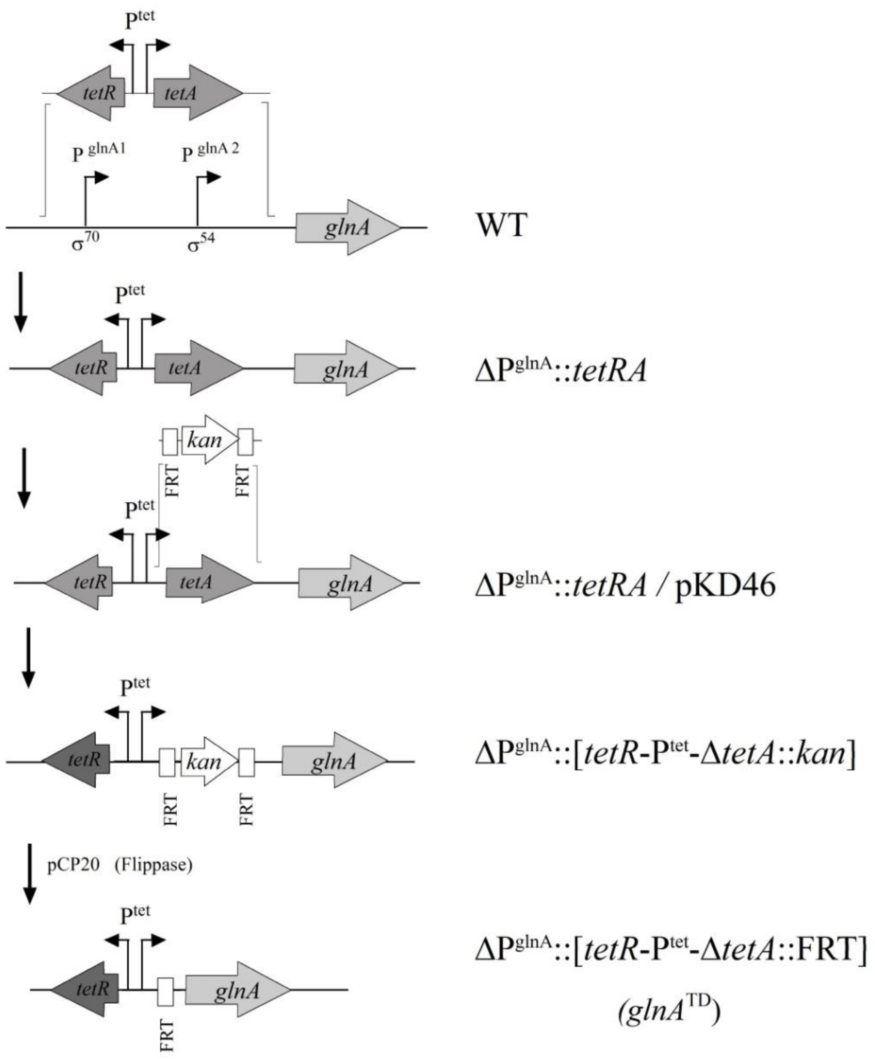

Supplementary Figure 4 . Construction of strains with a tetracycline-inducible system. Briefly, using the Red-Swap allelic replacement technique, the promoter of *glnA* gene was replaced by *tetRA* cassette. Then, *tetA* was exchanged by the KAN resistance cassette, encoded in the plasmid pKD4. Finally, the resistance to KAN was excised with the help of Flippase enzyme encoded in the plasmid pCP20. The final constructs contained *glnA* genes under control of the P^{tet} promoter. Similar constructs were created replacing the original promoters of *ompF* and STY4173. We called these constructs as tetracycline-dependent (TD).

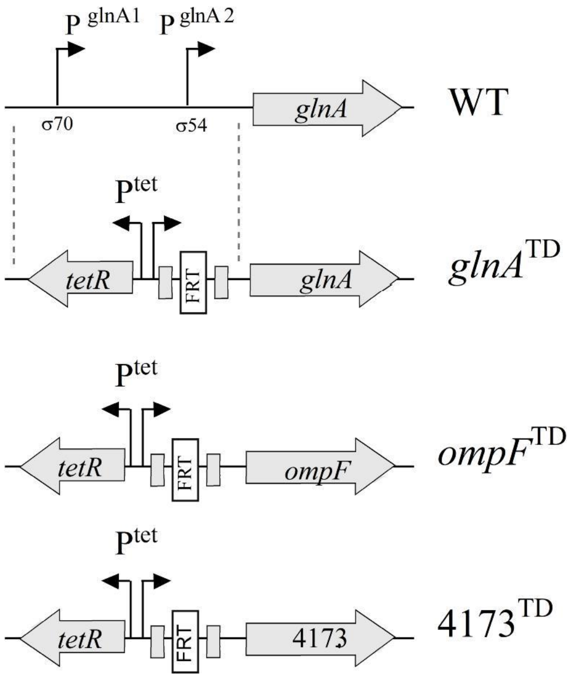

Supplementary Figure 5. Expression of *glnA*, *ompF*, and STY4173 using the tetracycline-inducible system. The mutants were called tetracycline-dependent (TD). Details regarding constructions of the TD mutants are shown in **Supplementary Figure 4**.

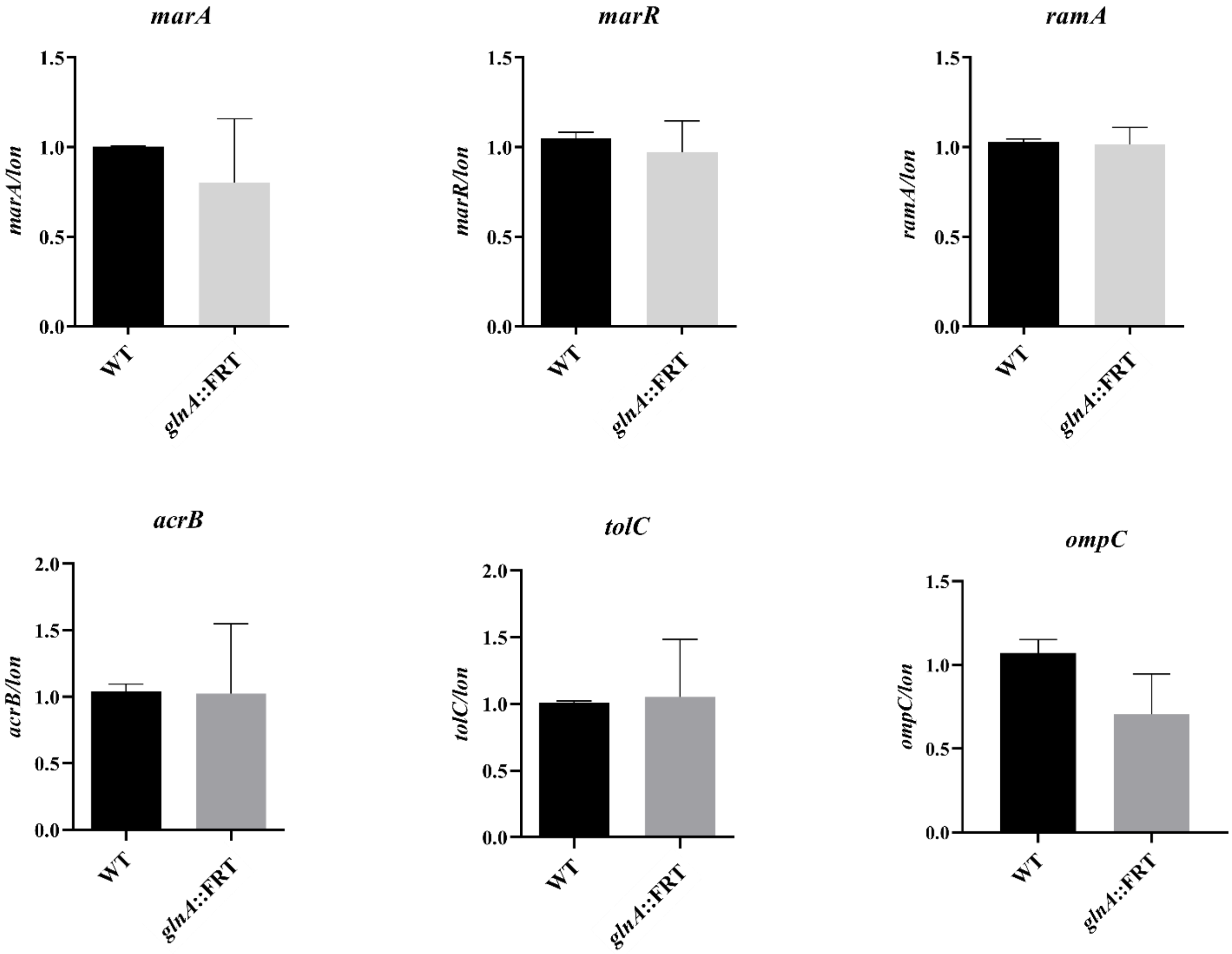

Supplementary Figure 6. Expression level of *marA, marR, ramA, acrB, tolC* and *ompC* transcripts in the mutant *glnA*::FRT compared to WT strain. Expression of transcripts was normalized to the expression of *lon* and compared to the expression of transcripts in the WT strain. No significant differences were observed calculated by Student's T-test. Results are average of at least 3 biological replicates.

1.2 Supplementary Tables

Supplementary Table 1. Primers used in this work

**Primer for mutant construction**	
aefA H1P1	TATTACAATTAGCGTTCTTATTATCCTGCTGTCCTGTCAGTGTAGGCTGGAGCTGCTTCG
aefA H2P2	GCCCTTAGCGTTATGCAGATGCACCTCAAGCTGGTTAAAGCATATGAATATCCTCCTTAG
glnA H1P1	GAGTACAAGTATGTCCGCTGAACACGTTTTGACGATGCTGTGTAGGCTGGAGCTGCTTCG
glnA H2P2	TAATATACGATTAAACGCTGTAGTACAGCTCAAACTCTACCATATGAATATCCTCCTTAG
glnG H1P1	GAATAGTCTGGGTCGTCGATGATGATAGTTCCATCCGTTGTGTAGGCTGGAGCTGCTTCG
glnG H2P2	TTATTAATCTTTACACTCGCGGATAATGTTTACTCCATTCCATATGAATATCCTCCTTAG
glnL H1P1	ATGCACTAAAATGGTGCAACCTTTTCCAGGAGACTGCTGATGTAGGCTGGAGCTGCTTCG
glnL H2P2	CCGCTGACCATCGGGTAGAACAGCGTATCCTGTAAATGGCATATGAATATCCTCCTTAG
micF HIP1	GGGTGTGTCTGAAAAAGGGTAAAAAAAAACCGAATGCGAAGCATCCGTGCAGGCTGGAGCTGCTTC
micF H2P2	TATATTCACGAAACTTTTAAAATCAACGGGTTAAATTGATGAAATCATATGAATATCCTCCTTAG
ompF H1P1	ATATAAAAAAACCAATGAGGGTAATAAATAATGATGAAGCTGTAGGCTGGAGCTGCTTCG
ompF H2P2	CCGCCTGATCGTCGGTGCCAACGTAGCTGGAGCTGTAGTC CATATGAATATCCTCCTTAG
H1tetR prom glnA FW	ACAAGTATTGCAGAGTCTCTTTGTGATCGCTTTCACGGAGGGAAAAAGGTTATGCTGCTT
H2tetA prom glnA RV	ACTTGTACTCTCCCGGATTGGTCATGGTCGGTCGTCGTGGATCATTTGGTGACGAAATAA
H1tetR prom ompF FW	TAATAAAATCATAGTGATAAAATAAAAATAATCAAATCAAGGAAAAAGGTTATGCTGCTT
H2tetA prom ompF RV	TATTTATTACCCTCATTGGTTTTTTTATATGACACCTGCC ATCATTTGGTGACGAAATAA
H1tetR prom 4173 FW	AGGTTGCCAGGTTTATACCAGTCAACGTGCGGGGGTTTTCGGAAAAAGGTTATGCTGCTT
H2tetA prom 4173 RV	AGAGCCCCCTGCCCAAAATATACGCAGCTCTCTCATAAAGATCATTTGGTGACGAAATAA
**Primer for mutant verification**	
KAN-2 FP-1 Forward Primer	ACCTACAACAAAGCTCTCATCAACC
R6KAN-2 RP-1 Reverse Primer	CTACCCTGTGGAACACCTACATCT
aefA comp FW	TGCTGGAGATGTATCAATTGTGTCCGACG
aefA comp RV	CGAAAAGGGCTTGAGCAAGGGTTGGCAC
glnA comp FW	CACAAACATCCTCCGCAAAC
glnA comp RV	CTTGCAGCAACGCGAAAT
glnG comp FW	AATTGAGCAAGTACTGCTGA
glnG comp RV	GCTGAACAGTCCCACCAGCG
glnL comp FW	AGCATAGATAGCGATGTGAC
glnL comp RV	CAGAGCGACGGCTTCATCAA
ompF comp FW	AGATGCCTGTCAGACACATAAA
ompF comp RV	CCGTCAATGCCGAGATAGTT
comp tetR prom glnA FW	ACTCGCTCTTGAGTTTCGGC

comp tetR prom glnA RV	TGGACGCATCGGGCATCAGC
comp tetR prom ompF FW	TAATAAAATCATAGTGATAAAATAAAAATAATCAAATCAAGGAAAAAGGTTATGCTGCTT
comp tetR prom ompF RV	CCCTGATACTGGATACCGAAAG
comp prom tetR 4173 FW	TGTTGCCATTACGGCGGACA
comp prom tetR 4173 RV	TAGGCGATGATAAAAGCGGT
**Primer for RT-qPCR**	
acrB RT FW	CGGATGAAGACCAGGGCGTATTC
acrB RT RV	CCCATGCTTTCAACGACACAAATG
dnaN RT FW	ACGTCGGCGACTTTATCTTTAC
dnaN RT RV	GCCCGCTTCCAGATGTTTAT
glnA RT FW	ACCGATGTTCGGCGATAAC
glnA RT RV	CTCAGACAGACCGGCATATTT
glnG RT FW	ACCGCGCATTCCGATTTA
glnG RT RV	GAGCGACGGCTTCATCAATA
glnL RT FW	GAAGTGACGCTGGTGATTGATAG
glnL RT RV	GCGCCATTTCGAGCAGTATAA
lon RT FW	CGCAGATGTGGCAATGACCG
lon RT RV	CGCTTCACAGGATGAATATCCAGATC
marA RT FW	AATGGCACCTGCAACGGATGTTTA
marA RT RV	ATGGTTCAGCGGCAGCATATACCGT
marR RT FW	AAAGTGCTGTCTGTCGATCTCGGCG
marR RT RV	TGAGCAAATACTCAAGCGTTGCCAC
micF RT FW	ACAGAATCTTCATTCGCAACTA
micF RT RV	GGTAAACAGACATTCAGAAGTGA
ompC RT FW	GACGCAGGTTCCTTCGATTAT
ompC RT RV	TAAAGTTGTCAGCGCCGTAG
ompF RT FW	GCGCCTATACCGATAACTACAT
ompF RT RV	CCCTGATACTGGATACCGAAAG
ramA RT FW	CGCTCAGGTTATCGACACGATTGTC
ramA RT RV	GACAAATATCATACACCTTCTGGTCGGTG
tolC RT FW	CGGTAACTCGGTCGGTACACGTAC
tolC RT RV	CTGCGGCAGCATCTTGATCTGGCGT

Supplementary Table 2. Zones of inhibition of *S*. Typhi EZ-Tn*5* mutants selected (mm)

	Inhibition haloes (mm)					
	S. Typhi STH2370					
Antimicrobial	WT	*recC*::EZ-Tn*5*	*cysE*::EZ-Tn*5*	*aefA*::EZ-Tn*5*	*dacC*::EZ-Tn*5*	*glnA*::EZ-Tn*5*
CIP	37.3	45.7	43.2	45.3	46.3	48.6
LVX	35.0	40.0	38.5	35.0	45.0	38.0
NAL	29.0	35.0	28.0	37.0	30.0	35.0
MEM	34.3	37.0	32.0	32.0	39.0	35.0
IPM	35.0	37.0	34.0	33.0	40.0	32.0
ETP	37.0	40.0	35.0	38.0	47.0	40.0
CRO	40.0	43.0	40.0	50.0	45.0	45.0
AMP	31.5	34.0	31.0	32.5	38.0	35.0
AMC	31.5	34.5	28.0	29.0	36.0	31.0
TMP	32.5	31.0	35.0	39.0	33.0	35.0
ERY	7.0	7.0	7.0	19.0	7.0	7.0
TET	29.0	31.5	27.0	29.0	39.0	34.0
GEN	27.0	32.0	28.0	28.0	36.0	30.0
CAM	34.0	38.5	35.0	43.0	45.0	40.0
ACR	18.0	20.0	18.0	35.0	18.0	18.0
MV	16.8	17.0	22.0	23.0	14.0	18.0
BZ	27.0	31.0	31.0	30.0	23.0	27.0
DC	7.0	7.0	7.0	7.0	7.0	7.0
PMB	29.0	7.0	26.0	26.0	32.0	31.0

ACR, acriflavine; AMC, amoxicillin/clavulanate; AMP, ampicillin; BZ, benzyl viologen; CRO, ceftriaxone; CAM, chloramphenicol; CIP, ciprofloxacin; DC, deoxycholic acid; ERY, erythromycin; ETP, ertapenem; GEN, gentamicin; IPM, imipenem; LVX, levofloxacin; MEM, meropenem; MV, methyl viologen; NAL, nalidixic acid; PMB, polymyxin B; TET, tetracycline; TMP, trimethoprim.
